# Supplementary material for: Antibody testing for COVID-19: A report from the National COVID Scientific Advisory Panel
Source: Wellcome Open Res. 2020 Jun 11;5:139. [Version 1] doi: 10.12688/wellcomeopenres.15927.1 (PMC7941096; doi:10.12688/wellcomeopenres.15927.1)
Supplement: Peer review history

# Peer review: Reviewer response for version 1

**DOI:** 10.21956/wellcomeopenres.17469.r42752

**Published:** 2021-03-08

**Authors:** Muki Shey

**Affiliations:** 1 Wellcome Centre for Infectious Disease Research in Africa (CIDRI-Africa) & Department of Medicine, University of Cape Town, Cape Town, South Africa

The article by Adams and colleagues “Antibody testing for COVID-19: A report from the National COVID Scientific Advisory Panel” compares the performance of “in-house” ELISA with 9 lateral flow Immunoassays (LFIA) from different companies, using samples from pre-pandemic period and RT-PCR-confirmed COVID-19 cases at different stages of the disease. The ELISA showed 85% sensitivity and 100% specificity. The LFIA show higher sensitivity and specificity compared with ELISA than with RT-PCR, and the performance of the LFIA were similar to each other. The authors conclude that ELISA performs better than LFIA for individual patient applications.

**Comments**

1. The authors describe that the ELISA used measured antibodies against the spike protein but not much info is available about the different LFIA and what parts of the virus may have been used to develop and optimize the antibody detection. This information is important to have a complete understanding of the comparison between the ELISA and the LFIA (Table 1).
2. Most recommended quarantine/isolation period (may be country-dependent) is 10-14 days from symptom onset and the authors describe up to 28 days as acute period. A justification may be necessary. Was this predefined, based on information around viral shedding or antibody kinetics (similar to data presented in Figure 2D)?
3. In line with Tables 1 and S4 and the false negative results, can the authors comment on the antibodies possibly targeting other parts of the virus other than spike proteins (especially for the ELISA since details of the LFIA are not available)?

Is the work clearly and accurately presented and does it cite the current literature?

Yes

If applicable, is the statistical analysis and its interpretation appropriate?

Yes

Are all the source data underlying the results available to ensure full reproducibility?

Partly

Is the study design appropriate and is the work technically sound?

Yes

Are the conclusions drawn adequately supported by the results?

Yes

Are sufficient details of methods and analysis provided to allow replication by others?

Yes

Reviewer Expertise:

Immunology and infectious Diseases.

I confirm that I have read this submission and believe that I have an appropriate level of expertise to confirm that it is of an acceptable scientific standard.

# Peer review: Reviewer response for version 1

**DOI:** 10.21956/wellcomeopenres.17469.r40226

**Published:** 2020-09-15

**Authors:** Yee Joo Tan, Kim Tien Ng

**Affiliations:** 1 Infectious Disease programme, Department of Microbiology and Immunology, National University Health System (NUHS), National University of Singapore, Singapore, Singapore; 2 Infectious Disease programme, Department of Microbiology and Immunology, National University Health System (NUHS), National University of Singapore & Institute of Molecular and Cell Biology (IMCB), A*STAR (Agency for Science, Technology and Research), Singapore, Singapore

This is a systematic study that compares a laboratory-based home-made ELISA serological assay for COVID-19 with nine commercially-available rapid lateral flow immunoassay (LFIA). The results presented are convincingly and important for the development/improvement of serological assays for COVID-19. It will be helpful if the authors can clarify the following points:

  

1. Page 4, Methods: 2nd paragraph, line 4: Acute (28 days).
  Query: How did the authors decide on the 28 days cut-off? Is this definition similar to other studies on COVID-19 seroconversion? It would be good to discuss and include citation.
2. Page 6, it was stated “We also considered performance relative to ELISA ( *Extended data*, Table S5, Figure S1), because the LFIA devices target the same antibodies.”
  Query: The viral protein used each of the LFIA device is not stated in the manuscript. Based on this sentence, are the authors stating that all of them use SARS-CoV-2 Spike residues 1-1208? If not, please state the viral protein or fragment used for each device.
3. Page 9, “… with animal data demonstrating that SARS-CoV-2 anti-spike IgG contributes to a proinflammatory response associated with lung injury in macaques.”
  Query: SARS-CoV-2 should be SARS-CoV.
4. Page 10, it was stated “We observed several LFIA false positives, which may have potentially resulted from cross-reactivity of non-specific antibodies (e.g. reflecting past exposure to other seasonal coronavirus infections).
  Query: Are these LFIA using the same viral protein or fragment? What is the sequence homology between SARS-CoV-2 and endemic coronaviruses for each of viral protein used?
5. Page 10, it was stated “In contrast to others, we did not find evidence of an association between disease severity and antibody titres”
  Query: Is there any difference in study design between this study and the published studies cited? Were the published studies measuring anti-Spike binding antibodies or were they measuring neutralizing antibodies? Are the sample size and distribution of cases (mild vs severe, etc) comparable? Please highlight any limitation that may explain the discrepancy.
6. For the simulations shown in Figure 4.
  Query: Please provide details on method/program as well as assumptions used.
7. The ELISA described by the authors is important and adds value to the development of serological assay for COVID-19. To briefly show the robustness of the assay, can authors provide information on (a) intra-assay variability, (b) inter-assay variability, (c) inter-operators variations and (d) the turnaround time i.e. from sample to result?

Is the work clearly and accurately presented and does it cite the current literature?

Partly

If applicable, is the statistical analysis and its interpretation appropriate?

Yes

Are all the source data underlying the results available to ensure full reproducibility?

Yes

Is the study design appropriate and is the work technically sound?

Yes

Are the conclusions drawn adequately supported by the results?

Yes

Are sufficient details of methods and analysis provided to allow replication by others?

Partly

Reviewer Expertise:

Molecular virology; Monoclonal antibody technology;

We confirm that we have read this submission and believe that we have an appropriate level of expertise to confirm that it is of an acceptable scientific standard.
